# Axenfeld–Rieger syndrome combined with ectropion uveae and pigment dispersion syndrome: A case report

**DOI:** 10.1097/MD.0000000000032869

**Published:** 2023-02-17

**Authors:** Yang Li, Jie Liu, Qingmei Tian, Xianzhen Ma, Yuhui Zhao, Hongsheng Bi

**Affiliations:** a Shandong University of Traditional Chinese Medicine, Jinan, PR China; b Affiliated Eye Hospital of Shandong University of Traditional Chinese Medicine, Jinan, PR China; c Shandong Provincial Key Laboratory of Integrated Traditional Chinese and Western Medicine for Prevention and Therapy of Ocular Diseases; Key Laboratory of Integrated Traditional Chinese and Western Medicine for Prevention and Therapy of Ocular Diseases in Universities of Shandong; Eye Institute of Shandong University of Traditional Chinese Medicine, Jinan, PR China.

**Keywords:** Axenfeld–Rieger syndrome, case report, ectropion uveae, pigment dispersion syndrome

## Abstract

In January 2021, we found one case of Axenfeld–Rieger syndrome combined with pigment dispersion syndrome (PDS), and this patient additionally manifested a symptom of ectropion uveae. The co-existence of both 2 syndromes is very rare and has not been reported in any literature yet.

**Case presentation:**

A 34-year-old female truck driver presented to our institution with a dimness of vision in her right eye. The patient had obvious posterior embryotoxons at bitamporal, and peripheral anterior synechia could be visualized by the slit lamp. The dispersion of pigment granules was observed behind the cornea. The pupil was slightly shifted upwards the nose, with 360° ectropion uveae. Gonioscopy revealed pigment accumulation on the trabecular meshwork. The patient underwent cataract surgery on her right eye, during which, flaky pigmentation around the posterior capsule was observed. These signs were consistent with Axenfeld–Rieger syndrome and PDS.

**Conclusions:**

We report a rare case of Axenfeld–Rieger syndrome with PDS and uveal eversion. Although the patient did not present with glaucoma, follow-up should be noted. Besides, the correlation between these 2 syndromes needs to be demonstrated by more cases or further evidence.

## 1. Introduction

Axenfeld–Rieger syndrome is an autosomal dominant disease characterized by an abnormal anterior segment with a prevalence estimated at 1 in 50,000 to 100,000 newborns.^[1]^ Pigment dispersion syndrome (PDS) is characterized by abnormal loss of iris pigment and dispersion of pigment granules throughout the anterior segment of the eye.^[2]^ The co-existence of both 2 syndromes is very rare and has not been reported in any literature yet. In January 2021, we found one case of Axenfeld–Rieger syndrome combined with PDS, and this patient additionally manifested a symptom of ectropion uveae (EU). The details of the diagnosis and treatment of this case are as follows.

## 2. Case presentation

**Figure 1.**
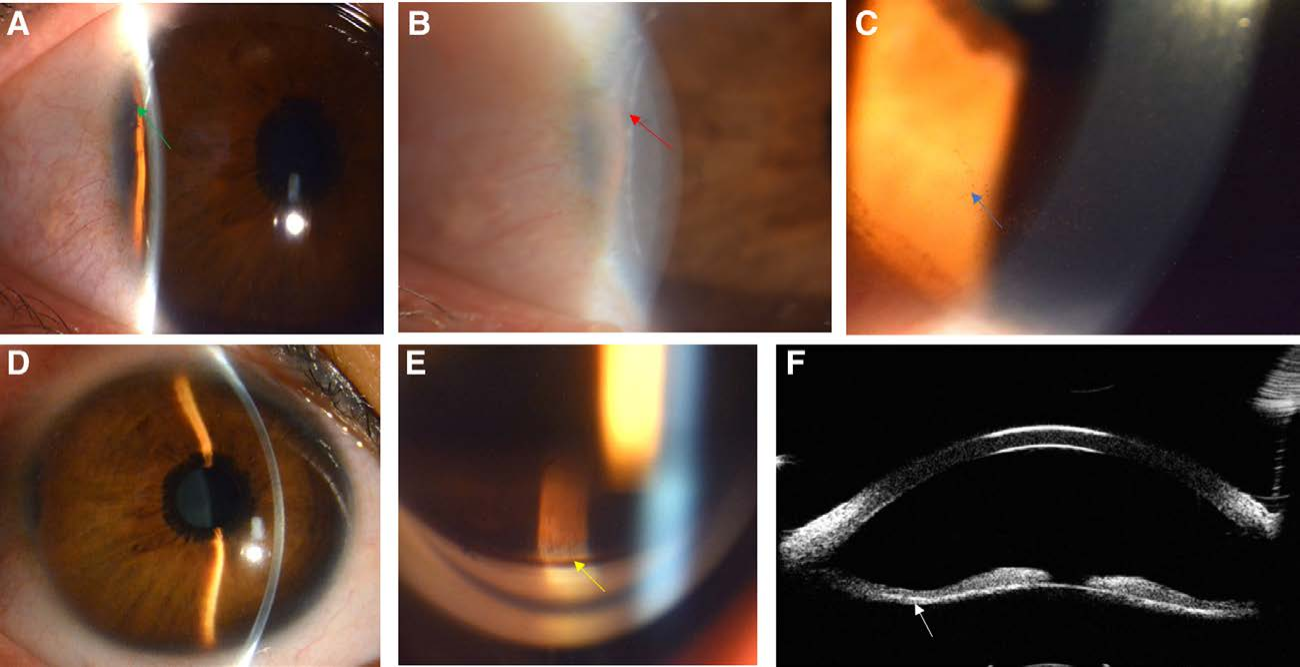
(A) Photograph showing peripheral anterior synechiae (green arrow). (B) Posterior embryotoxon (red arrow) in the right eye. (C) The dispersion of pigment granules (blue arrow) was observed behind the cornea. (D) The anterior chamber of the eye was deep, the mid-peripheral part of the iris was sunken backwards, the uvea around the pupil edge was turned over. (E) Gonioscopy showing that the pigment granules were heavily dispersed over the trabecular meshwork with color like soy sauce (yellow arrow). (F) UBM showing that the iris was fine and sunken backwards, closely adhering to the len (white arrow). UBM = ultrasound biomicroscopy.

A female truck driver of 34 years old came to the Eye Hospital Affiliated to Shandong University of Traditional Chinese Medicine on January 24, 2021, due to a loss of right vision for 1 year. She was diagnosed with “cataracts” in the local hospital 2 years ago and received left cataract surgery there without special treatment for her right eye. She had poor vision in the left eye since childhood and denied a history of hypertension, diabetes, and a family history of glaucoma or hereditary disease. The general examinations showed fasting blood glucose and glycosylated hemoglobin levels of 13.2 mmol/L and 9.8% respectively, without any other significant abnormality. For eye examinations, the right visual acuity was 20/40 (+3.75/−0.25 × 110) without any improvement following correction, the left visual acuity was 20/125 and 20/100 (+1.25/−0.25 × 133) following correction, and the intraocular pressures were 19 mm Hg in both eyes. Slit-lamp examination revealed the conjunctiva was not hyperemic, a curved white line (posterior embryotoxon) was shown behind the corneal limbal at bitamporal, peripheral anterior synechia, the dispersion of pigment granules was observed behind the cornea (Fig. 1A–C), the anterior chamber of the eye was deep, the mid-peripheral part of the iris was sunken backward, the uvea around the pupil edge was turned over (Fig. 1D), and the pupil was slightly shifted upwards the nose, the cortex of right eye was opaque and of the artificial lens in the left eye was right positioned. Both pupil diameters were about 4 mm following mydriasis. Gonioscopy was conducted for both eyes with results showing that the anterior chamber angle was closed at bitamporal and was open at other sides, the pigment granules were heavily dispersed over the trabecular meshwork with color like soy sauce (Fig. 1E), and ultrasound biomicroscopy (UBM) showed that the iris was fine and sunken backward, closely adhering to the lens (Fig. 1F). The axis oculi of right eye and left eye were 23.6 mm and 24.49 mm, and the average corneal curvatures of right eye and left eye were 39.78D and 40.24D, and the results of the rest of exploration including fundoscopy and visual field were strictly normal. The results of ophthalmologic diagnosis included complicated cataract (right eye), PDS (both eyes), Axenfeld–Rieger syndrome (both eyes), congenital EU (both eyes), intraocular lens implantation (left eye), and amblyopia (left eye). The medical consultation recommended regulation of blood glucose for 1 week and then we performed cataract surgery on her right eye, during which, flaky pigmentation around the posterior capsule was observed. On day 1 after the operation, the intraocular pressure of the right eye was 31.7 mm Hg, Timolol was dropped, and the intraocular pressure was dropped to normal 2 days later. The visual acuity was 20/20 and the intraocular pressure was normal on week 1 after the operation.

## 3. Discussion and conclusions

The Axenfeld–Rieger syndrome is mostly autosomal dominant inheritance, mainly manifested as peripheral anterior synechiae attached in the angle of the anterior chamber and posterior embryotoxon, which may be combined with thinning of stroma iridis. Glaucoma is the most serious complication of Axenfeld–Rieger syndrome, and 50% of patients were reported to develop a glaucoma.^[3]^ This patient had obvious posterior embryotoxons at bitamporal, and peripheral anterior synechia could be visualized at the slit lamp. The pupil was slightly shifted upwards the nose, and UBM showed a fine echo to the iris. This patient denied a family history and this case would be considered sporadic.

EU is a rare non-progressive anomaly characterized by the presence of iris pigment epithelium on the anterior surface of the iris stroma, which is usually not inherited. Since EU can be associated with systemic diseases, such as neurofibromatosis type 1, Prader–Willi syndrome,^[4]^ we carefully made a general checkup on this patient, including her teeth, bellybutton, skin and intelligence level, etc, without any abnormality found. Glaucoma is a common complication of EU, while this patient’s intraocular pressure was normal without any injury to the optic nerve and visual field.

The PDS results from the sunken back mid-peripheral part of the iris contacting and rubbing with the zonular fibers, resulting in depigmentation of the iris epithelium so that the pigment granules are dispersed over the corneal endothelium, lens, and trabecular meshwork, etc via the aqueous humor circulation. The open-angle glaucoma secondary to pigment dispersion is called pigmentary glaucoma. Young population with high myopia is susceptible. It has been found that the higher the degree of myopia, the younger the age at which the optic nerve damage is brought by glaucoma. In this case, the patient was a young female who had normal axis oculi and slightly flat curvatures and did not manifest as high myopia, but her anterior chamber was deep, and anomalous iridozonular contact was found by the slit lamp and UBM, which provided an anatomical basis for pigment dispersion. PDS is often accompanied by the Kruckenberg spindle on the corneal endothelium, and although this patient had posterior corneal pigmentation, it was not a typical spindle. The pigmentation on the corneal endothelium and the pigments of uniform densities over the trabecular meshwork confirmed the pigment dispersion. Her stroma iridis was thinned, the dilator pupillae muscle was weakened, and in addition, she was also accompanied by diabetes (diagnosed following admission). Therefore, the pupil dilation was not large enough and the periphery of the lens was difficult to be examined before the operation so the abnormal pigment dispersion could not be found. The flaky pigment dispersion was found around the posterior capsule of the lens following intraoperative implantation of a pupil dilator to successfully phacoemulsificate the nucleus and cortex. Since studies reported a risk of 35 to 50% for developing pigmentary glaucoma in patients with PDS,^[5]^ we instructed the patient for regular follow-up, and although her intraocular pressures, fundus, and visual fields were normal.

To our knowledge, the Axenfeld–Rieger syndrome combined with EU and PDS was very rarely reported, and the correlation between these 2 syndromes needs to be demonstrated by more cases or further evidence. Unfortunately, we had limitations in our report, one of which was the lack of genetic information for the case. Although this patient suffered from these 2 syndromes and EU, fortunately, there had been no significant abnormality in her intraocular pressures, fundus, and visual fields so far. Therefore, besides the cataract surgery, we did not conduct any other special treatment, while we explained to the patient details of her state of illness and informed her of regular follow-ups in her lifetime to avoid possible damage brought by glaucoma.

The patient was satisfied with the treatment. She said the doctor found the abnormality in her right eye through careful examination and carefully made a general checkup on her teeth, bellybutton, skin, and intelligence level, etc, without any abnormality. Doctors gave her a full account of her illness, and she said she would follow up regularly throughout her life to avoid possible damage from glaucoma.

## Author contributions

**Conceptualization:** Hongsheng Bi.

Data curation: Yang Li.

Formal analysis: Yang Li, Jie Liu.

Investigation: Yang Li, Qingmei Tian, Xianzhen Ma, Yuhui Zhao.

Methodology: Hongsheng Bi.

Resources: Jie Liu, Hongsheng Bi.

Validation: Qingmei Tian, Xianzhen Ma, Yuhui Zhao.

Visualization: Yang Li, Jie Liu, Qingmei Tian, Xianzhen Ma, Yuhui Zhao.

Writing – original draft: Yang Li.

Writing – review & editing: Jie Liu.
